# CLINICAL TRIALS USING MUSIC IN DIVERSE COMMUNITIES OF OLDER ADULTS

**DOI:** 10.1093/geroni/igae098.3937

**Published:** 2024-12-31

**Authors:** Tara Rose

**Affiliations:** University of Southern California, Los Angeles, California, United States

## Abstract

Music is being integrated into health care services and nonpharmacological treatment options as part of physical health and well-being programs for older adults. When health care professionals and older adult providers try to locate successful programs to initiate, they look to clinicaltrials.gov as a resource to find the most rigorous and robust programs implemented. In large US cities, such as Los Angeles, where there are multiple ethnic populations with specific cultural music interests, it is important to have knowledge of culturally specific interventions in order to create evidence-based programs for multiple community members. The purpose of this review is to analyze music programs and interventions for African American, Asian American, and Latino/Hispanic older adults to better understand emerging best practices. A search of all trials registered in the clinicaltrials.gov was conducted using key words describing these ethnic populations and also the word “music.” Of the 146 studies generated, 15 met the eligibility criteria, with all studies enrolling older adults along with other age groups. Studies were conducted across four regions of the US, 53% in Northeast, 20% South, 13% Midwest, and 13% West. Music interventions for specific medical conditions (20% dementia and 27% other diseases) and mental health/well-being (53%) were the primary foci in studies. Data on intervention types, demographics, and methodology will be presented. Results to date suggest that more research is needed on best practices in music interventions for specific cultural groups and that new approaches would benefit cities and communities, especially in areas with diverse multicultural populations.

